# Clinical and angiographic characteristics of patients with STEMI and confirmed diagnosis of COVID-19: an experience of Tanta University Hospital

**DOI:** 10.1186/s43044-020-00103-y

**Published:** 2020-10-06

**Authors:** Ahmed Alaarag, Timoor Hassan, Sameh Samir, Mohamed Naseem

**Affiliations:** Department of Cardiology, Tanta University, Tanta, Egypt

**Keywords:** STEMI, Primary PCI, COVID-19, High thrombus burden, TIMI flow

## Abstract

**Background:**

Patients with established cardiovascular diseases have a poor prognosis when affected by the coronavirus disease 2019 (COVID-19). Also, the cardiovascular system, especially the heart, is affected by COVID-19. So we aimed to evaluate the angiographic and clinical characteristics of COVID-19 patients presented by ST-elevation myocardial infarction (STEMI).

**Results:**

Our retrospective study showed that STEMI patients with COVID-19 had elevated inflammatory markers with mean of their CRP (89.69 ± 30.42 mg/dl) and increased laboratory parameters of thrombosis with mean D-dimer (660.15 ± 360.11 ng/ml). In 69.2% of patients, STEMI was the first clinical presentation and symptoms suggestive of COVID-19 developed during the hospital stay; about one third of patients had a non-obstructive CAD, while patients with total occlusion had a high thrombus burden.

**Conclusion:**

STEMI may be the initial presentation of COVID-19. A non-obstructive CAD was found in about one third of patients; on the other hand, in patients who had a total occlusion of their culprit artery, the thrombus burden was high. Identification of the underlying mechanism responsible for the high thrombus burden in these patients is important as it may result in changes in their primary management strategy, either primary PCI, fibrinolytic therapy, or a pharmaco-invasive strategy. Furthermore, adjunctive anticoagulation and antiplatelet therapy may need to be revised.

## Background

Although most of the coronavirus disease 2019 (COVID-19) patients often have mild symptoms or may be asymptomatic, some patients may present with more serious symptoms, including pneumonia and acute respiratory distress syndrome. Patients with traditional cardiovascular risk factors like hypertension, diabetes, obesity, male sex, and those with established cardiovascular diseases are considered high-risk patients with increased mortality and morbidity when affected by COVID-19 [1, 2].

The cardiovascular system, especially the heart, is affected by the COVID-19. This affection could be through direct myocardial injury due to hypoxia, hemodynamic instability, thrombosis due to hypercoagulability, and myocarditis. Moreover, the cytokine storm resulted from systemic inflammation could lead to plaque rupture [3].

Viral pneumonia and influenza are associated with increased risk of acute myocardial infarction up to six folds [4] the virus utilize angiotensin-converting enzyme 2 (ACE2) receptor to enter the cells. The ACE2 is expressed in the vascular endothelium; thus, the viremia could directly destabilize plaques and causing type 1 myocardial infarction (MI); moreover, COVID-19 can induce type 2 MI by increasing myocardial demand [5].

### Aim of work

In the current study, we aim to evaluate the angiographic and clinical characteristics of COVID-19 patients presented by ST-elevation myocardial infarction (STEMI).

## Methods

### Study population

The present study retrospectively included 26 patients with STEMI and confirmed diagnosis of COVID-19. All patients treated with a primary percutaneous coronary intervention (PPCI) during the period from March 2020 till June 2020.

The study was approved by the local ethical committee. As our study is retrospective and data were collected after patients discharged from the hospital, there is no consent for participation in our study.

Confirmed acute STEMI was defined based on the presence of typical anginal pain more than 20 min, associated with new left bundle branch block or new ST-elevation at the J-point in two contiguous leads with the cut-point: > 1 mm in all leads other than leads V2–V3 where the following cut-points apply: > 2 mm in men > 40 years, > 2.5 mm in men <40 years, or > 1.5 mm in women regardless of age. The diagnosis was confirmed by the elevation in troponin levels [6].

Patients who have symptoms suspicious for COVID-19, e.g., progressively worsening shortness of breath, cough, fever, body aches, and unexplained hypoxemia were referred for chest CT without contrast. Laboratory tests (complete blood count, C-reactive protein (CRP), and serum ferritin) were done. COVID-19 was confirmed with reverse transcription-polymerase chain reaction assays.

### Angiographic procedure

Coronary angiography and percutaneous coronary intervention were done through the femoral or radial approach. All patients received the following regimen: (1) ticagrelor 180 mg initial dose followed by a maintenance dose of 90 mg twice daily or clopidogrel 600 mg loading dose orally followed by a maintenance dose of 75 mg/day if ticagrelor is contraindicated, (2) aspirin 300 mg followed by 75–100 mg/day, and (3) during the procedure patients received unfractionated heparin (100 IU/kg), the dose was reduced to (70 IU/kg) in case of administration of glycoprotein IIb/IIIa inhibitor (eptifibatide).

Thrombolysis in myocardial infarction (TIMI) flow rate [7] was assessed before and at the end PPCI.

Obstructive coronary artery disease was defined as a ≥ 50% stenosis in the coronary artery lumen [8].

Thrombus burden (TB) was graded (G) as G0 = no thrombus, G1 = possible thrombus, G2 = small [greatest dimension ≤ 1/2 vessel diameter (VD)], G3 = moderate (> 1/2 but < 2VD), G4 = large (≥ 2VD), G5 = unable to assess TB due to vessel occlusion. In patients with G5 thrombus, burden score was calculated after passage of wire or small non-inflated balloon through the lesion [9].

### Echocardiographic evaluation

Echocardiographic evaluation was performed using the commercially available GE Vivid 7 echocardiograph with 2.5 MHz transducer. LV ejection fraction (EF) was evaluated (using the biplane method of disks) [10].

### Statistical analysis

All statistical studies were carried out using Statistical Package for Social Sciences software (SPSS 20.0 for Windows, SPSS Inc., Chicago, IL). The quantitative variables are expressed as mean ± standard deviation (SD). Normally distributed scale variables were expressed as mean ± standard deviation. Non-normally distributed variables were expressed as median and range. Categorical variables were expressed in numbers and percentages.

## Results

Twenty-six patients with STEMI and confirmed diagnosis of coronavirus disease 2019 (COVID-19), who underwent PPCI, were retrospectively included in the study.

### Baseline clinical characteristics (Table 1)

Male patients represented 69.2% (*n* = 18), while female patients represented 30.8% (*n* = 8). The mean age of the patients were 57.37 years ± 8.75 with range 28–73 years. Regarding traditional risk factors the incidence of diabetes, hypertension, current smoking status, and dyslipidemia were 38.5% (*n* = 10), 42.3% (*n* = 11), 50.0% (*n* = 13), 38.5% (*n* = 10), respectively. Three patients (11.5%) had history of prior percutaneous coronary intervention (PCI) and 1 patient (3.8%) had history of prior CABG.

**Table 1:** Basal clinical characteristics

	N	Range	Mean	SD
**Age**	26	28–73	57.73	8.75
**O2 saturation (%) at presentation**	26	80–98	93.00	3.94
**BMI (Kg/m2)**	26	2233	27.69	2.90
**Serum creatinine (mg/dl)**	26	0.7–2.2	1.36	0.43
**CRP (mg/dl )**	26	18–200	89.69	30.42
**D-dimer (ng/ml)**	26	100–2000	660.15	360.11
**SBP (mmHg)**	26	80–140	119.o	14.0
**HR (beat/min)**	26	60–120	81.0	12.0
			***N***	**%**
**Sex**	Male		18	69.2
	Female		8	30.8
**Diabetes**	Yes		10	38.5
	No		16	61.5
**Hypertension**	Yes		11	42.3
	No		15	57.5
**Current smoker**	Yes		13	50
	No		13	50
**Dyslipidemia**	Yes		10	38.5
	No		16	61.5
**Leukopenia**	Yes		13	50
	No		13	50
**Relative lymphopenia**	Yes		20	76.9
	No		6	23.1
**Killip class**	I		16	61.5
	II		6	23.1
	III		3	11.5
	IV		1	3.8
**Prior PCI**	Yes		3	11.5
	No		23	88.5
**Prior CABG**	Yes		1	3.8
	No		25	.96.2

*PCI* percutaneous coronary intervention, *CABG* coronary artery bypass graft, *SD* standard deviation

The mean heart rate (beat/min), systolic blood pressure (mmHg), oxygen saturation%, and EF% of our patients were 81, 119, 93, and 44, respectively. Sixteen patients (61.5%) presented by Killip class I, 6 patients (23.1%) by Killip class II, 3 patients (11.5%) by Killip class III, and 1 patient by cardiogenic shock Killip class IV.

Levels of CRP (C-reactive protein) (mg/dl) and D-dimer (ng/ml) were elevated (89.69 ± 30.42 and 660.15 ± 360.11, respectively). Leukopenia and relative lymphopenia were present in 50% and 76.9% of patients, respectively.

### Clinical presentation and in-hospital outcome (Table 2)

Fifteen patients (57.7%) presented by anginal chest pain as the main presenting symptom and eleven patients (42.3%) presented with angina equivalent symptoms.

**Table 2:** Clinical presentation and the outcome

	*N*	Range	Mean	± SD
**EF%**	26	25–56	44.0	8.0
		***N***	**%**	
**Main presenting symptoms**	Chest pain	15	57.7	
	Angina equivalent symptoms	11	42.3	
**Other symptoms suggestive of COVID-19 at presentation**	Yes	10	38.5	
	No	16	61.5	
**Site of infarction in ECG**	Inferior	11	42.3	
	Anterior	9	34.6	
	Inferior and right	1	3.8	
	Inferior and lateral	1	3.8	
	Inferior and posterior	1	3.8	
	Lateral	2	7.7	
	LBBB	1	3.8	
**Outcome**	Re-infarction	3	11.5	
	Discharge	22	84.6	
	Death	4	15.4	

*EF* ejection fraction, *LBBB* left bundle branch block

In 69.2% of patients (*n* = 18), STEMI was the first clinical presentation and symptoms suggestive of COVID-19 developed during the hospital stay. The remaining eight patients (30.8%) had clinical manifestations suggestive of COVID-19 at presentation. None of the study patients had COVID-19 test results available at the time of PPCI. The mortality rate of our patients was 15.4% (*n* = 4 patients).

One of the four dead patients had an extensive anterior STEMI and total occlusion of his left anterior descending artery with failure of recanalization and no-reflow. The patient developed cardiogenic shock on the second day and had asystole at the time of death.

The second patient had an extensive anterior STEMI and developed contrast-induced nephropathy and became anuric on the second day. He had severe electrolyte disturbance and had asystole at the time of death.

The third patient had inferior myocardial re-infarction after successful revascularization. His coronary angiography showed an acute stent thrombosis; the patient developed heart block and had asystole at the time of death.

The fourth patient had extensive anterior STEMI. He had a successful PCI; however, after 2 days, he suddenly developed ventricular fibrillation and died without any evidence of new ischemic events.

### Angiographic characteristics (Table 3)

Regarding the localization of STEMI 42.4% of patients (*n* = 11) had inferior STEMI; 34.6% of patients (*n* = 9) had anterior STEMI; 7.7% of patients (*n* = 2) had lateral STEMI; inferior and right, inferior and lateral, and inferior and posterior STEMI were present in 3 patients of patients (3.8% one for each localization); and 3.8% (*n* = 1) presented by LBBB.

**Table 3:** Angiographic characteristics

	*N*	Range	Mean	± SD
**Time from onset of symptoms to FMC (min)**	26	120–1560	638.08	407.08
**Time from FMC to wire crossing (min)**	23	45–90	66.96	12.50
		***N***	**%**	
**Culprit artery**	No	3	11.5	
	RCA	9	34.6	
	LAD	8	30.7	
	LCX	4	15.4	
	LM	1	3.8	
	D1	1	3.8	
**Number diseased vessels**	Single	13	50	
	Two	8	30.8	
	Three	5	19.2	
**TIMI flow before PCI**	0	13	50	
	1	4	15.4	
	2	5	19.2	
	3	4	15.4	
**TIMI flow after PCI**	0	2	7.7	
	1	3	11.5	
	2	6	23.1	
	3	7	26.9	
	N/A	8	30.8	
**Thrombus burden grade**	1	2	7.7	
	2	7	26.9	
	3	5	19.2	
	4	10	38.5	
	5	2	7.7	
**Luminal % stenosis of culprit lesion**	≤ 50%	8	30.7	
	> 50 – 99%	5	19.2	
	100%	13	50	
**Acute stent thrombosis**		4	15.4	

*FMC* first medical contact, *PCI* percutaneous coronary intervention, *TIMI* thrombolysis in myocardial infarction, *N/A* not applicable

Time from symptoms onset to first medical contact (FMC) and from FMC to wire crossing were 638.08 ± 407.08 and 66.96 ± 12.50 min, respectively.

Eight patients did not have obstructive coronary artery. The culprit artery cannot be identified in 3 patients (11.5%) with TIMI 3 flow was present in all vessels, and five patients (19.2%) had a culprit artery with a non-obstructive lesion.

RCA was the culprit vessel in 34.6% of patients (*n* = 9), LAD culprit vessel in 30.7% of patients (*n* = 8), LCX in 15.4% of patients (*n* = 4), and LM and diagonal branches were the culprit in one patient for each (3.8%).

Thirteen patients had TIMI flow zero at the diagnostic coronary angiography, 15.4% (*n* = 4) had TIMI flow one, and 19.2% (*n* = 5) had TIMI flow two, and TIMI flow three was present in 15.4% of patients (*n* = 4). In two patients (7.7%), the procedure ended by no-reflow and failure of recanalization.

Two patients (7.7%) had thrombus burden grade one with the uncertainty of their culprit lesion, seven patients (26.9%) had thrombus burden grade two, five patients (15.3%) had thrombus burden grade three, ten patients (38.5%) had thrombus burden grade four, and two patients (7.7%) had thrombus burden grade 5 with failed recanalization (no-reflow).

Six patients (23.1% ) had chest pain after PCI, of whom 3 (11.5%) patients had ST-segment re-elevation (re-infarction) and coronary angiography showed stent thrombosis in 4 (15.4% ). The remaining 2 patients did not have any significant changes compared to the previous procedures.

Half of the patients (*n* = 13) had single-vessel disease without any significant lesions in the non-culprit vessels, 30.8% (*n* = 8) had two vessel disease, and 19.2% (*n* = 5) had three vessel disease.

## Discussion

The novel coronavirus, severe acute respiratory syndrome coronavirus 2 (SARS-CoV-2), which causes coronavirus disease 2019 (COVID-19), is highly contagious in the community and has resulted in a global pandemic [11].

Myocardial injury, due to underlying ischemia, acute thrombotic occlusion, or myocarditis, is reported in 7–28% of hospitalized COVID-19-positive patients and is associated 7–10 with higher mortality [12, 13].

Many countries, including Egypt, have instituted measures to limit transmission of the virus. Egypt was implementing a partial night-time curfew during the study time, and this may explain the delay we noticed in the presentation of patients with STEMI in the present study. Atypical symptoms and patients’ avoidance of hospitals for fear of contracting COVID-19 infection may be other factors that enhanced the delayed presentation of STEMI.

STEMI, as the first clue of COVID-19, was found in 61% of our patients. The different angiographic findings of patients ranged from an inability to find a culprit lesion to total occlusion of the culprit artery with a high thrombus burden may be explained by the different mechanisms the SARS-CoV-2 affects the heart.

A direct myocardial injury can occur as ACE2 receptors, which are the site at which the SARS-CoV-2 attach to enter the cell are expressed in 7.5% of myocytes [14]. Also, myocardial injury can be caused by hypoxia and demand-supply mismatch caused by hemodynamic effect of sepsis [15]. Myocardial injury in these conditions may be associated with non-significant coronary lesions on coronary angiography. SARS-CoV-2 can elicit an intense pro-inflammatory and cytokine storm leading to vascular inflammation and plaque rupture associated with a hypercoagulable state and platelets activation [16, 17], also disseminated intravascular coagulopathy (DIC) was found in 71.4% (15/21) of non-survivors with COVID-19 and 0.6% (1/162) of survivors [18].

Also, COVID-19 infection is associated with a pro-thrombotic state. The occurrence of venous thrombo-embolic complications appears to be an important manifestation of the disease and one which is related to disease severity and outcome [19]. The participation of these mechanisms may explain the high thrombus burden in patients of COVID-19 and presented by STEMI.

Guo et al. [12] showed in their retrospective single-center study that patients with cardiovascular co-morbidities had a more cardiac injury when get infected by (SARS-CoV-2). 54.5% of patients with cardiovascular co-morbidities had an elevated troponin level compared to 13.2% of those without; this may explain the relatively high mortality (15.4%) in our patients.

Also, Guo et al. [12] found that there was a higher incidence of malignant arrhythmia in patients admitted to intensive care units with elevated troponin 11.5% compared to 5.2% for those with normal troponin. In our study, we had one death due to ventricular fibrillation 2 days after a successful PCI without any evidence of new ischemic events.

Reports about the clinical and angiographic findings and outcomes of patients with COVID-19 and STEMI are still very few. Stefanini et al. [20] evaluating the COVID-19 patients with STEMI in Lombardy, Italy, showed that 78.6% of patients presented with chest pain and 21.4% presented with dyspnea, while we showed that angina equivalent is the presenting symptom in 42.3% of patients. They also showed that 39.3% had non-obstructive CAD, which is close to our finding (30.8% had non-obstructive CAD).

Hamadeh et al. [21] in a study performed in 4 countries in COVID-19 patients with STEMI primary PCI was the treatment strategy in 24% of patients, while fibrinolytic therapy was the treatment in 76% of patients. Mortality was 26% compared to 14.4.% in our series, and stent thrombosis was found in 21% while in our study, it was 15.4%.

From these findings, we recommend that management of STEMI in the context of a COVID-19 outbreak should balance between the safety of health care providers and adequate patient treatment.

## Conclusion

STEMI may be the initial presentation of COVID-19. A non-obstructive CAD was found in about one third of patients; on the other hand, patients who had a total occlusion of their culprit artery, the thrombus burden was high. Identification of the underlying mechanism responsible for the high thrombus burden in these patients is important as it may result in changes in their primary management strategy, either primary PCI, fibrinolytic therapy, or a pharmaco-invasive strategy. Furthermore, adjunctive anticoagulation and antiplatelet therapy may need to be revised.

### Limitations

Our study is retrospective and had a small number of patients; we recommend a national multicenter study to address this issue in a wide range of patients.

## Data Availability

The datasets used and or analyzed during the current study are available from the corresponding author on reasonable request.

## Abbreviations

COVID-19: Coronavirus disease 2019
ACE2: Angiotensin-converting enzyme 2
MI: Myocardial infarction
STEMI: ST-elevation myocardial infarction
PPCI: Primary percutaneous coronary intervention
TIMI: Thrombolysis in myocardial infarction
TB: Thrombus burden
VD: Vessel diameter
EF: Ejection fraction
FMC: Thrombus burden
SARS-CoV-2: Severe acute respiratory syndrome coronavirus 2
